# Characterization of extracellular vesicles by capillary zone electrophoresis: A novel concept for characterization of a next-generation drug delivery platform

**DOI:** 10.1016/j.jpha.2024.101004

**Published:** 2024-05-23

**Authors:** Aleksandra Steć, Andrea Heinz, Szymon Dziomba

**Affiliations:** a Department of Toxicology, Faculty of Pharmacy, Medical University of Gdansk, 80-416 Gdansk, Poland; b LEO Foundation Center for Cutaneous Drug Delivery, Department of Pharmacy, University of Copenhagen, 2100 Copenhagen, Denmark

**Keywords:** Capillary electrophoresis, Characterization, Electrolyte, Extracellular vesicles, Heterogeneity

## Abstract

Extracellular vesicles (EVs) are a part of a cell-to-cell communication system of prokaryotic and eukaryotic organisms. Their ability to penetrate biological barriers and to transfer molecules between cells shows their potential as a novel class of drug delivery platform. However, because of the great heterogeneity of EVs and the complexity of biological matrices from which they are typically isolated, reliable quality control procedures need to be established to ensure their safety for medical use. According to current recommendations, quantification of EVs, confirmation of their identity, and purity assessment require the use of several analytical techniques, including particle-size distribution analysis, proteomics, and electron microscopy, making the characterization process demanding. Capillary electrophoresis (CE) has recently emerged as an alternative tool for EV characterization. In this study, the available literature on this novel concept for EV characterization was reviewed. Its performance was critically evaluated and compared with currently used methods. The utility of CE in the quality control of EV-based medicines was discussed.

## Highlights

• The analysis of EVs with CE was reviewed.
• Theoretical and practical aspects of electrophoresis of vesicles were discussed.
• CE was compared with reference methods for quality control of EVs.
• Discussion of future perspectives in this area was conducted.

## Graphical abstract

**Figure:**
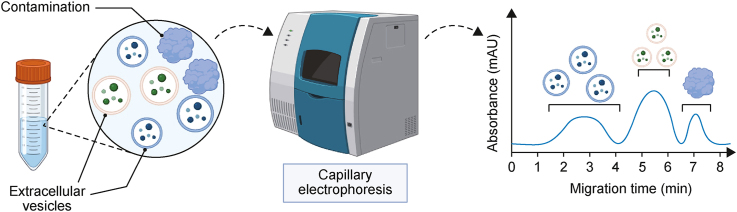

## 1 Introduction

**Fig. 1:**
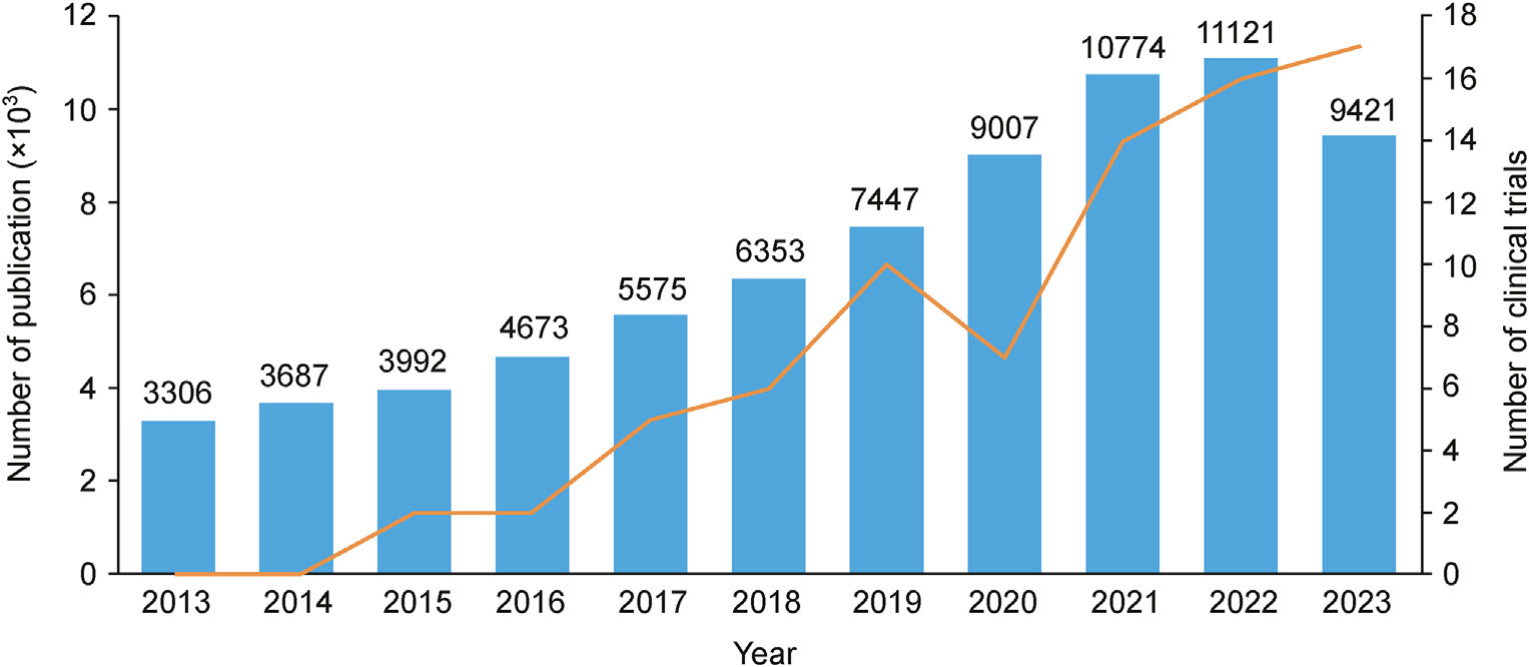
Number of articles published on extracellular vesicles (EVs) (blue bars) and EV-related clinical trials (orange trace) registered in the last ten years. Data were obtained from the Web of Science (search formula: “extracellular vesicles” or “exosomes” or “membrane vesicles”) and clinicaltrials.gov (search formula: “extracellular vesicles”) in December 2023.

Extracellular vesicles (EVs) are spherical nanostructures and microstructures secreted by living cells. EVs are carriers of cellular components, including proteins, lipids, and nucleic acids. Among its many functions, cell-to-cell communication is commonly highlighted as the most essential role of EVs. This includes gene transfer between bacterial cells [1], stimulation of cancer cell proliferation and migration [2], and modulation of the immune response of the organism [3]. The latter property is currently widely studied for EV application in regenerative medicine and inflammation-driven diseases, including neurological, cardiovascular, and pulmonary diseases [4]. The ability of EVs to penetrate biological barriers and transfer a great variety of molecules makes them a promising drug delivery platform. Furthermore, EVs can target certain organs or can be engineered for this purpose [5]. For instance, EV modification has shown to increase the efficiency of drug delivery (reduction of the required therapeutic dose) and improve EV circulation kinetics [6,7]. The biological and medical importance of EVs explains an increase in the number of scientific papers published on this topic and clinical trials registered in the last decade (Fig. 1). However, this dynamically developing field of science is not devoid of technical challenges related to the isolation of EVs and the quality control of EV isolates [8,9].

Human-derived mesenchymal stem cells are currently the most important source of clinically relevant EVs [4,10]. Plant material, nonhuman milk, and prokaryotic organisms are generally considered to be alternative sources that have significant implementation potential [10]. For instance, bacterial EV-based vaccine has been successfully used to prevent meningitis B for over 30 years [11]. The isolation of EVs from diverse sources requires the development of individual purification and preconcentration strategies. The isolation of EVs is a multistep process that usually combines two or three isolation techniques, such as differential ultracentrifugation (UC) and/or density gradient centrifugation (DGC), size-exclusion chromatography (SEC), precipitation, and immunoprecipitation [8,9,12,13]. During the isolation, EVs are separated from soluble components of the matrix, protein aggregates, lipoparticles, viruses, and cell debris. Moreover, the isolate may contain various types of EVs, and the isolation itself may be a source of process-related impurities. All these potential impurities make the isolation of EVs demanding [[13], [14], [15]]. Characterization of obtained isolates include quantitation of EVs, assessment of purity, detection of impurities, and confirmation of the identity of EVs [14,15].

This study critically evaluates the potential of capillary electrophoresis (CE) as a tool for the characterization of EV isolates and quality control of EV-based medicinal products. The entire available literature published since 2018 was reviewed [16]. The work was focused on the basics of the EV electromigration phenomenon, aspects related to vesicle detection, the problem of adsorption to capillary walls, and issues associated with the background electrolyte (BGE) composition. Finally, CE was compared with well-established techniques used for EV characterization and its utility in the pharmaceutical analysis of this novel class of biological drugs was discussed.

## 2 CE for the characterization of EV isolates

### 2.1 Principles of CE

CE is a high-performance separation technique. In CE, the separation occurs in liquid medium under high electric field (usually 200−1,000 V cm^−1^) when difference in the electrophoretic mobility of sample components is reached [17]. Capillary zone electrophoresis (CZE) is the basic mode of CE, in which the electrophoresis is conducted in a simple electrolyte solution. In such a BGE, the electrophoretic mobility (*μ*) of the analyte is dependent on its charge (*q*) to size (diameter, *d*) ratio and the dynamic viscosity (*η*) the BGE, as described in Eq. 1:

(1) $$\mu \;=\;\frac{q}{3\pi d\eta }$$

The separation process is routinely conducted in silica capillaries. Small diameters of the capillaries (preferentially 25−100 μm) limit not only the heat generated during current conduction but also the required sample volume (generally 5−20 nL per analysis). The utilization of simple electrolytes as BGEs and a relatively small capillary inner volume make the technique cost-effective, result in negligible consumption of chemicals (less than 100 μL per run), and meet the assumptions of green chemistry.

The great advantage of CZE is its ability to simultaneously separate various types of analytes, including inorganic ions, small molecules, proteins, nucleic acids, and nano- and micro-particles [18]. CE has become established, among other fields, in genetics (Sanger sequencing) and the pharmaceutical industry, where it is currently mostly used for the quality control of proteins and biological drugs [19].

### 2.2 Behavior of particles in an electric field

#### 2.2.1 Electric double layer (EDL)

**Fig. 2:**
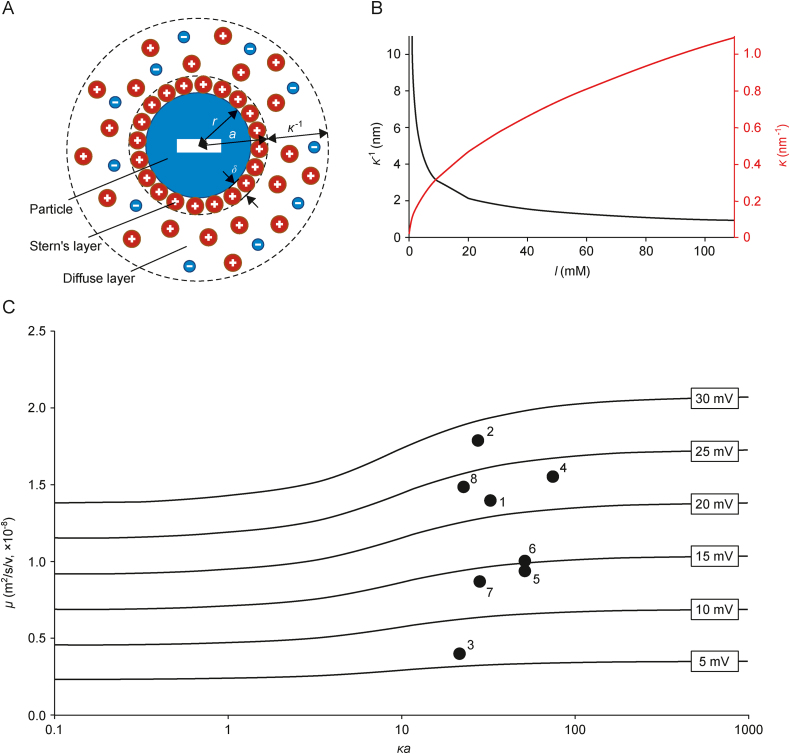
Characterization of particle behavior in liquid medium under electric field conditions. (A) Electric double layer (EDL) formed on the surface of a negatively charged particle of the radius (*r*). The first layer is formed by the strongly adsorbed ions (Stern's layer). The second layer is diffusive. The size of both Stern's and diffusive layers is defined by thickness (*δ*) and Debye length (*κ*^−1^), respectively. (B) Influence of ionic strength (*I*) on the size of the diffuse layer (*κ*^−1^) and its reciprocal. (C) Relation between electrophoretic mobility (*μ*) and *κa* according to the Ohshima approximation of Henry's function (Eqs. (3), (5)). The presented description is simplified and does not include the relaxation effect that substantially contributes to the migration when *ζ* > 40–50 mV. Dots represent the estimated mobility and *κa* of EVs reported in different publications (the legend and calculated values are shown in Table 1). *a*: particle radius; *κ*: Debye parameter.

EVs are spherical, micro- and nanosized particles exhibiting a net-negative charge. When dispersed in liquid media such as the BGE used for CE analysis, the surface charge of the particles is screened by counterions present in the solution. The counterions create an immobile (in relation to the surface of the particles) layer of ions adsorbed to the surface of the particles (Stern's layer) and a layer of loosely associated ions, susceptible to electric and thermal forces (a diffuse layer) (Fig. 2A). Stern's and diffuse layers form the EDL. The thickness (*δ*) of Stern's layer relates to the diameter of the adsorbed ions and, together with the solid particle radius (*r*), constitutes the particle radius a, which is the sum of *r* + *δ* (Fig. 2A). *a* is considered to be identical to the Stokes radius [20]. The thickness of the diffuse layer is defined as Debye length (*κ*^−1^) and is dependent on the ionic strength (*I*) of the liquid under constant temperature conditions (Fig. 2B). In a 1:1 electrolyte, the reciprocal of the Debye length (Debye parameter, *κ*) is defined in Eq. 2:

(2) $$\kappa \;=\;\sqrt{\frac{2000\;e^{2}N_{A}I}{\varepsilon_{r}\varepsilon_{o}kT}}$$

where *e* denotes elementary electric charge; *N*_A_ means Avogadro's number; *ε*_r_ and *ε*_*o*_ mean relative electric permittivity of electrolyte and vacuum, respectively; *k* mean Boltzmann constant; and *T* means temperature.

While *a* is characteristic of certain EVs, the size of *κ*^−1^ (and its reciprocal, *κ*) is easily tunable by the BGE composition. Investigating *κ*^−1^ as a function of ionic strengths *I* typically used in CE, it becomes evident that *κ*^−1^, i.e., the size of the diffuse layer, substantially changes and has a major influence on the size of the EV and its EDL, particularly when diluted buffers are used (*I* → 0; Fig. 2B). In this context, it needs to be pointed out that the application of buffers with low ionic strengths (*I* < 10 mM) is not recommended for CE analysis due to the low buffering capacity of such electrolytes. Furthermore, the steep slope of the curves (Fig. 2B) indicates that the use of BGEs with low *I* values results in a great variability of *κ*^−1^ of the dispersed particles (and therefore, the *μ* (Fig. 2C); see Section 2.2.2) due to electrolyte depletion, leading to low robustness of the method.

#### 2.2.2 Electrophoretic mobility

The existence of EDL has a considerable impact on the electrophoretic mobility *μ* of a particle as screening ions co-migrate with particles. This fact has several consequences such as the occurrence of retardation and relaxation effects, which complicate the description of the electrophoretic motion of a particle. The former phenomenon results from the electrophoretic migration of screening counterions in the opposite direction as compared with migrating particles. The latter is related to the deformation of EDL (EDL polarization) under an electric field. As the relaxation effect is meaningful only if *ζ* > 40–50 mV [21,22], which is not expected for EVs under typical experimental conditions [23], it will not be discussed in detail. Instead, an excellent review article discussing this topic is recommended [20]. Nevertheless, the contribution of the EDL to the electrophoretic properties of migrating particles is reflected in the fact that *μ* is a function of *κa*, which is described by Henry's equation (Eq. 3) [24]:

(3) $$\mu \;=\;\frac{2\varepsilon_{r}\varepsilon_{o}\zeta }{3\eta }{ƒ}_{H}\left(\kappa a\right)$$

where *ε*_*r*_ and *ε*_*o*_ denote the relative electric permittivity of the electrolyte and vacuum, respectively, and *ζ* denotes the *ζ*-potential.

For relatively big particles under high ionic strength (high *κa*), Henry's function (*f*_H_(*κa*) in Eq. (3) is approaching 1.5 (Smoluchowski equation) (Eq. 4):

(4) $$\mu =\frac{\varepsilon_{r}\varepsilon_{o}\zeta }{\eta }$$

Smoluchowski equation is suitable for the description of electrophoresis of microvesicles (diameter > 1 μm), assuming their rigidness. As these structures are soft and deformable in nature, Eq. 4 is insufficient for the precise characterization of their electrophoretic motion. However, no report on the CE of microvesicles has been published to date, and thus, this issue is out of the scope of this study.

Several approximations of Henry's function have been developed to date [20]. The semi-empirical formula proposed by Ohshima [22] provides good accuracy (relative error less than 1%) and can be applied to characterize μ of objects displaying physicochemical properties similar to EVs (*ζ* < 40 mV; *κa* > 10) (Eq. 5):

(5) $${ƒ}_{H}\left(\kappa a\right)\;=\;\frac{2}{3}\left[1\;+\;\frac{1}{2{\left(1\;+\;\frac{2.5}{\kappa a(1+2e^{-\kappa a})}\right)}^{3}}\right]$$

The relation between μ and *κa* described by Eqs. (3), (5) is shown in Fig. 2C, which indicates that the *μ* of EVs is dependent on the size of the vesicles only in a strictly defined region (≈1 < *κa* < 100). In the extreme ranges of the *κa* parameter, the *μ* value reaches a plateau, and size-dependent separation is not possible. Although the size-dependent separation of EVs has been undeniably confirmed in neither of the reviewed studies, data shown in Table 1 [[25], [26], [27], [28], [29], [30], [31]] and Fig. 2C indicate that the applied CE conditions were appropriate for such separation. Notably, the greatest difference of *μ* as a function of κa can be reached when *κa* ≈ 3–11 (the steepest slope of the curve). Such values may be achieved for small vesicles (10–70 nm), when *I* of buffers ranges from about 10 to 100 mM. Thus, the separation of small EVs (such as exosomes) from bigger EVs should be achievable. The impact of the composition and *I* of the BGE is further discussed in Section 2.3.

**Table 1:** Physical parameters calculated with data from different publications. Mode values obtained by nanoparticles tracking analysis (NTA) were used for the calculation of particle radius (*a*).

Sample	ID^a^	*μ* (m^2^/V/s)	*a* (nm)	*I* (mM)	*κ*^−1^ (nm)	*κ* (nm^−1^)	*κa*	Refs.
*Pectobacterium* sp. culturing medium	1	−1.40 × 10^−8^	59.0	28	1.81	0.55	32.3	[[25], [26], [27]]
CHO cells culturing medium	2	−1.78 × 10^−8^	50.0	28	1.81	0.55	27.6	[28]
*Citrus limon* juice	3	−4.02 × 10^−9^	40.0	28	1.81	0.55	22.0	[29]
Bovine milk	4	−1.55 × 10^−8^	76.5	90	1.01	0.99	75.6	[30]
Pony plasma	5	−9.46 × 10^−9^	53.0				52.4	
Pony serum	6	−1.01 × 10^−8^	53.0				52.4	
Human serum	7	−8.61 × 10^−9^	29.0				28.7	
Human serum	8	−1.48 × 10^−8^	37.0^b^	36	1.60	0.63	23.2	[31]

a Listed ID numbers were used to present data in Fig. 2C.

b *a* in this case was estimated based on *Z*-average value (dynamic light scattering, DLS). *μ*: electrophoretic mobility; *I*: ionic strength; *κ*^−1^: Debye length; *κ*: Debye's parameter. CHO: Chinese hamster ovary.

As regards proteins (and other biological components) present in biological fluids, it needs to be highlighted that these take part in the formation of Stern's and diffuse layers on the surface of EVs. In this form, they can be co-isolated together with the vesicles forming a so-called “protein corona” [32]. It was demonstrated that proteins affect the physicochemical and biological properties of vesicles [33]. Although no such experiments have been conducted in CE, a considerable impact of protein corona on μ can be assumed.

#### 2.2.3 Membrane-related effects

**Fig. 3:**
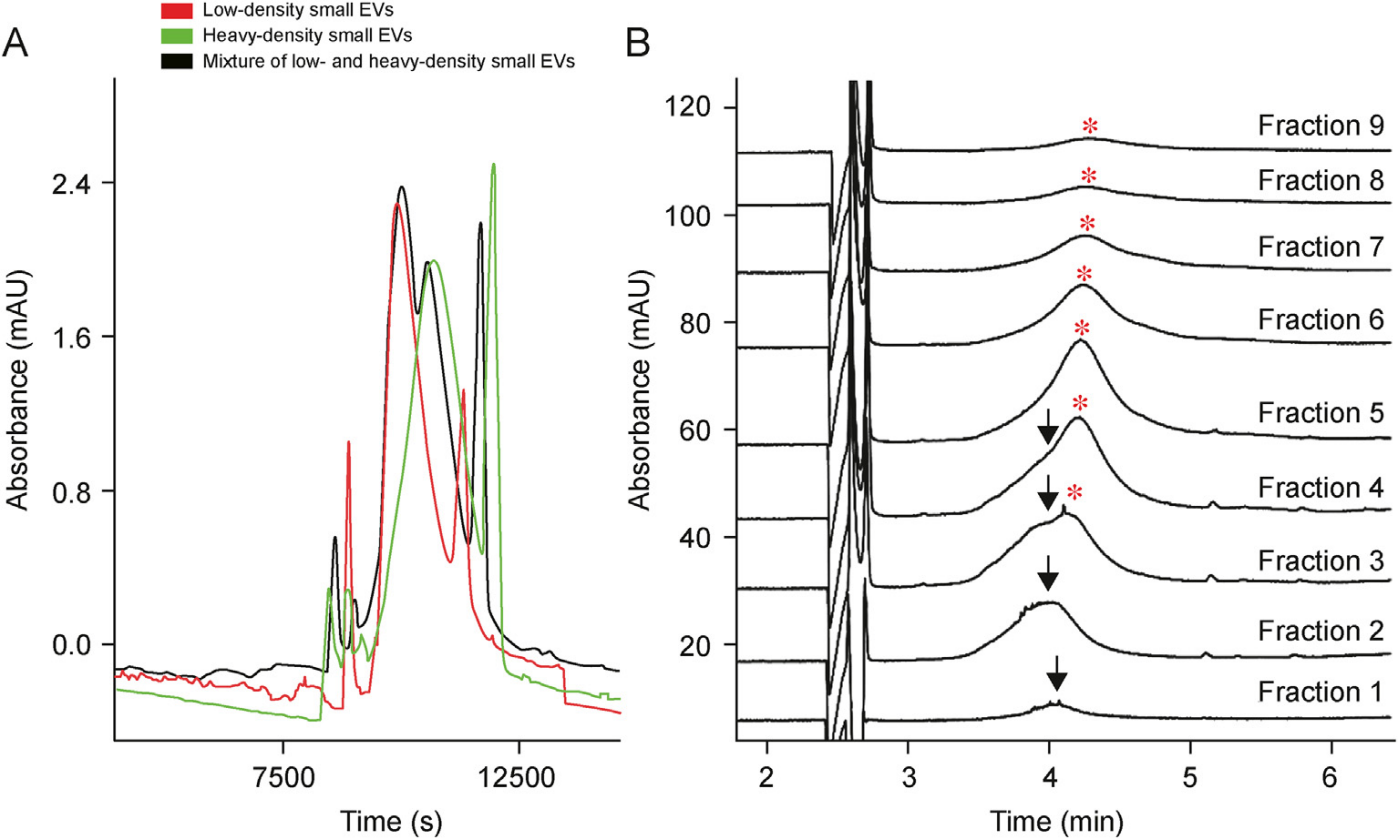
Capillary electrophoresis (CE) separation of extracellular vesicle (EV) subpopulations. (A) Capillary zone electrophoresis (CZE) separation of low-density small EVs (red trace), heavy-density small EVs (green trace), and their mixture (black trace). The fractions were obtained through density gradient centrifugation (DGC) of human urine samples [16]. (B) CE analysis of fractions obtained through DGC from green fluorescent protein (GFP)-modified *Pectobacterium odoriferum Car1* culture media [25]. Arrows and asterisks indicate signals generated by two subpopulations of EVs. Reprinted from Refs. [16,25] with permission.

Lan et al. reported the electrophoretic separation of two subpopulations of EVs using CE (Fig. 3A) [16]. The isolates were obtained from human urine samples through DGC, which implies various density and cargo of separated vesicles. No size differences were observed between these fractions.

Similar observations were made in a study focusing on bacterial outer-membrane vesicles (OMVs) (Fig. 3B) [25]. CE analysis of the fractions obtained through DGC revealed two populations of EVs featuring various *μ*. Neither nanoparticle tracking analysis (NTA) nor transmission electron microscopy (TEM) analysis revealed any difference between these fractions. Furthermore, the EVs in all the analyzed fractions were fluorescent due to the secretion of bacteria overproducing the green fluorescent protein (GFP). Thus, separation cannot be attributed to various sizes of the EVs or significant structural differences of the separated vesicles, but can be explained by the membrane-related effects (capacitive effect, polarization of the membrane, and deformability of the vesicles) instead [25].

**Fig. 4:**
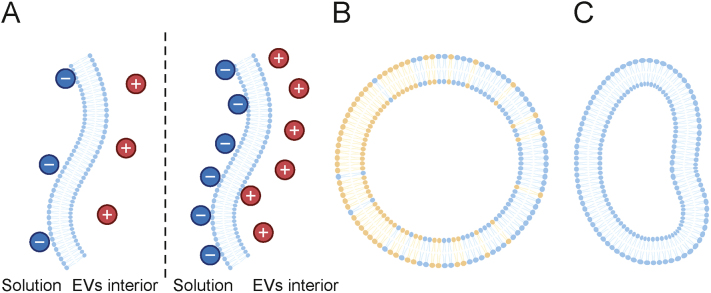
Membrane-related effects expected to occur during capillary electrophoresis (CE) separation of extracellular vesicles (EVs). (A) The capacitive effect results from the impact of the cargo of EVs on the external surface potential. The influence is dependent on the differences in pH value, ionic strength (*I*), and chemical composition of solutions on the two sides of the membrane. (B) Polarization of the membrane may be observed due to the fluid nature of the membrane. Local accumulation of chargeable components of the membrane may occur and lead to the uneven distribution of surface potential. (C) Deformation of the vesicle may occur due to the fluidity of the membrane and the presence of external forces.

Membrane-related effects are dependent on the chemical composition of EVs and have been reported for synthetic liposomes, i.e., particles structurally similar to EVs [34,35]. The capacitive effect results from the potential difference between the two leaflets of a bilayer membrane [34]. Consequently, the surface charge on one leaflet affects the charge on the other side (Fig. 4A). In the case of EVs, the capacitive effect is reflected in the *ζ* value of a vesicle altered by its cargo. The polarization effect arises from membrane fluidity. In an electric field, chargeable components of the membrane (e.g., phosphatidic acid) migrate and cumulate locally, leading to uneven charge distribution on the particle surface [35] (Fig. 4B). Although the experimental verification of the capacitive and polarization effects in the case of EVs would be very demanding, their presence is highly probable. In this context, it is necessary to mention that the mathematical formulas describing the electrophoretic motion of a particle (Eqs. (3), (4), (5)) are valid only for spherical objects. Thus, deviations from calculated values are expected because of the deformability of EVs (Fig. 4C). Considering the fact that membrane stiffness is dependent on several factors such as the chemical composition of the membrane as well as the temperature and density of vesicle-secreting cells, it becomes evident that a precise description of EV migration in an electric field may be challenging [36].

### 2.3 Impact of BGE

EV dispersions are subject to physical phenomena such as agglomeration, aggregation, and sedimentation [37]. In an electric field, the probability of aggregation is substantially increased due to the distortion of the EDL and interparticle interactions [38]. According to the Derjaguin-Landau-Verwey-Overbeek (DLVO) theory, the reduction of aggregation can be achieved by providing a high surface charge to the surface of the particle, which electrostatically stabilizes the dispersion [39]. This charge can be delivered to negatively charged particles using low-molar (low *I*) inorganic buffers, consisting of a buffering co-ion and a small, singly charged counterion (e.g., sodium). The application of the latter minimizes the screening of charge on the particle surface (high *ζ*). The borate buffer that was used to analyze EVs isolated from human urine samples and cell culture media met these criteria (Table 2) [16,[25], [26], [27], [28], [29], [30], [31],[40], [41], [42], [43], [44]]. Although the choice of the BGE composition was not discussed in the referred studies, borate buffer was often chosen as a BGE component owing to its ability to complex *cis*-diol bonds [45]. This property can be used to improve the solubility or separation efficiency of some molecules, including sugars, antibodies, and nucleic acids. The latter ones have been simultaneously separated from EVs in the study by Dou et al. [40]. In another study, the use of a 10-mM borate buffer (pH 9.0) was found to be sufficient for the separation of subpopulations of small EVs isolated from human urine samples (Fig. 3A) [16].

**Table 2:** Summary of extracellular vesicles (EVs) isolates characterized by capillary electrophoresis (CE).

Origin	Isolation methodology	BGE and I	Detection and capillary	Short summary	Refs.
Urine	Low-speed centrifugation, UC, and DGC	Borate buffer (pH 9.0); *I* = 20 mM	UV with uncoated fused silica capillaries (50 μm i.d. × 50 cm total capillary length)	● CE separation of EV subpopulations isolated with DGC	[16]
Bacteria culturing medium (*Pectobacterium* sp.)	Low-speed centrifugation, filtration, and UF in combination with UC (direct UC, iodixanol cushion UC, or DGC)	BTP/Gly (pH 9.5); *I* = 20 mM	UV with uncoated fused silica capillaries (50 μm i.d. × 30.2 cm total capillary length)	● Impact of isolate quality on the correlation between EV quantification results obtained by CE, BCA, and NTA ● CE separation of EV subpopulations isolated with DGC	[25]
Bacteria culturing medium (*Pectobacterium* sp.)	Low-speed centrifugation, filtration, and UC	CZE: BTP/Gly (pH 9.5); *I* = 20 mM	UV with uncoated fused silica capillaries (50 μm i.d. × 30.2 cm total capillary length)	● Correlation between CE and protein content assay test applied for EV quantitation ● Identification of macromolecular aggregates by CE	[26]
		Isotachophoresis: LE: Tris/POPSO (pH 8.6); *I* = 4 mM TE: Tris/AMPSO (pH 8.0); *I* = 11 mM	UV with poly(DMA-GMA-MAPS)-modified capillaries (50 μm i.d. × 30.2 cm total capillary length)		
Bacteria culturing medium (*Pectobacterium* sp.)	Low-speed centrifugation, filtration, UF, and iodixanol cushion UC	BTP/Gly (pH 9.5); *I* = 20 mM	UV and LIF with uncoated fused silica capillaries (50 μm i.d. × 30.2 cm total capillary length)	● Direct CE-LIF analysis of OMVs secreted by bacteria overexpressing GFP	[27]
CHO cells culturing medium	Low-speed centrifugation, UF, and SEC	BTP/Gly (pH 9.5); *I* = 20 mM	UV with uncoated fused silica capillaries (50 μm i.d. × 30.2 cm total capillary length)	● Monitoring of EV secretion by CHO cells overexpressing various proteins ● Isolation process monitoring with CE	[28]
*Citrus limon* juice	Low-speed centrifugation, filtration, UF, SEC, and precipitation	BTP/Gly (pH 9.5); *I* = 20 mM	UV and LIF with uncoated fused silica capillaries (50 μm i.d. × 30.2 cm total capillary length)	● Monitoring of the entire EV isolation method development with CE ● Correlation between EV quantification results obtained by CE, BCA, and NTA ● Staining of vesicle-encapsulated nucleic acids with SYBR Gold to confirm the identity of EVs	[29]
Bovine milk, pony plasma, pony serum, and human plasma	Low-speed centrifugation, filtration, UF, UC, DGC, SEC, and immunoaffinity chromatography	Tris/CHES (pH 8.4); *I* = 50–150 mM	LIF with uncoated fused silica capillaries (50 μm i.d. × 60 cm total capillary length)	● EV staining with CFDA-SE ● Quantification of EVs stained with CFDA-SE	[30]
Human blood	Low-speed centrifugation, PEG precipitation, and commercial vesicles	Tris/boric acid (pH 7.9) + 0.5% (*m*/*V*) HPC + 0.1% (*m*/*V*) SDS; *I* ca. 40 mM	CE-UV with silica capillaries (75 μm i.d. × 55 cm total capillary length)	● Adsorption of EVs to capillary walls	[31]
Cells culturing medium (MDA-MB-231, NCIH-1975, and CCL-119)	Low-speed centrifugation and UC	Borate buffer (pH 9.2); *I* = 50 mM	LIF with uncoated fused silica capillaries (75 μm i.d. × 59.1 cm total capillary length)	● CE quantification of EVs based on RNA content (YOYO-1 staining of nucleic acids released by lysed vesicles)	[40]
Bacteria culturing medium (*Pectobacterium* sp.)	Low-speed centrifugation, filtration, UF, and iodixanol cushion UC	BTP/Gly (pH 9.5); *I* = 8–40 mM Borate buffer (pH 9.3); *I* = 8–40 mM	UV with uncoated fused silica and poly(DMA-GMA-MAPS)-modified capillaries (50 μm i.d. × 30.2 or 60.2 cm total capillary length)	● Assessment of capillary coating on EVs adsorption during CE analysis ● Inhibition of electrophoretic migration of EVs by relaxation and membrane-related effects	[41]
Bovine milk, pony plasma, pony serum, and human serum	Low-speed centrifugation, filtration, sucrose DGC, UF, UC, SEC, and precipitation on magnetic beads	Tris/CHES (pH 8.4); *I* = 50–150 mM	LIF with uncoated fused silica capillaries (50 μm i.d. × 60 cm total capillary length)	● Assessment of isolation method performance	[42]
Exosomes (COLO-1 cells)	Commercial vesicles	PBS (0.5×; pH 7.4); *I* ca. 80 mM	UV with uncoated fused silica capillaries (50 μm i.d. × 35 cm total capillary length)	● Off-line coupling of AF4 and CE for separation and quantification of EVs ● separation of exosomes from selected blood components (common contaminants)	[43]
Culturing medium (HeLa cells)	Low-speed centrifugation, precipitation	HEPES/NaOH (pH 8.5); *I* ca. 20 mM	CEIA-LIF with uncoated fused silica capillaries (50 μm i.d. × 60 cm total capillary length)	● Indirect determination of exosomes with anti-CD-63 antibodies	[44]

BGE: background electrolyte; *I*: ionic strength; UC: ultracentrifugation; DGC: density gradient centrifugation; UV: ultraviolet; CE: capillary electrophoresis; EV: extracellular vesicles; UF: ultrafiltration; BTP: bis(tris(hydroxymethyl)methylamino)propane; Gly: glycine; BCA: bicinchoninic acid assay; NTA: nanoparticles tracking analysis; CZE: capillary zone electrophoresis; LE: leading electrolyte; POPSO: piperazine-*N*′,*N*′-bis(2-hydroxypropane sulphonic acid; TE: terminating electrolyte; AMPSO: *N*-(1,1-dimethyl:2:hydroxyethyl)-3-amino-2-hydroxypro: panesulfonic acid; DMA: *N*,*N*-dimethylacrylamide; GMA: glycidyl methacrylate; MAPS: [3-(methacryloyl-oxy)propyl]trimethoxysilane; LIF: laser-induced fluorescence; OMVs: outer-membrane vesicles; GFP: green fluorescent protein; CHO: Chinese hamster ovary; SEC: size-exclusion chromatography; CHES: *N*-cyclohexyl-2-aminoethanesulfonic acid; CFDA-SE: carboxyfluorescein diacetate succinimidyl ester; PEG: polyethylene glycol; HPC: hydroxypropyl cellulose; SDS: sodium dodecyl sulfate; PBS: phosphate-buffered saline; A4F: asymmetrical flow field-flow fractionation; HEPES: 4-(2-hydroxyethyl)-1-piperazineethanesulfonic acid; CEIA: CE immunoassay.

**Fig. 5:**
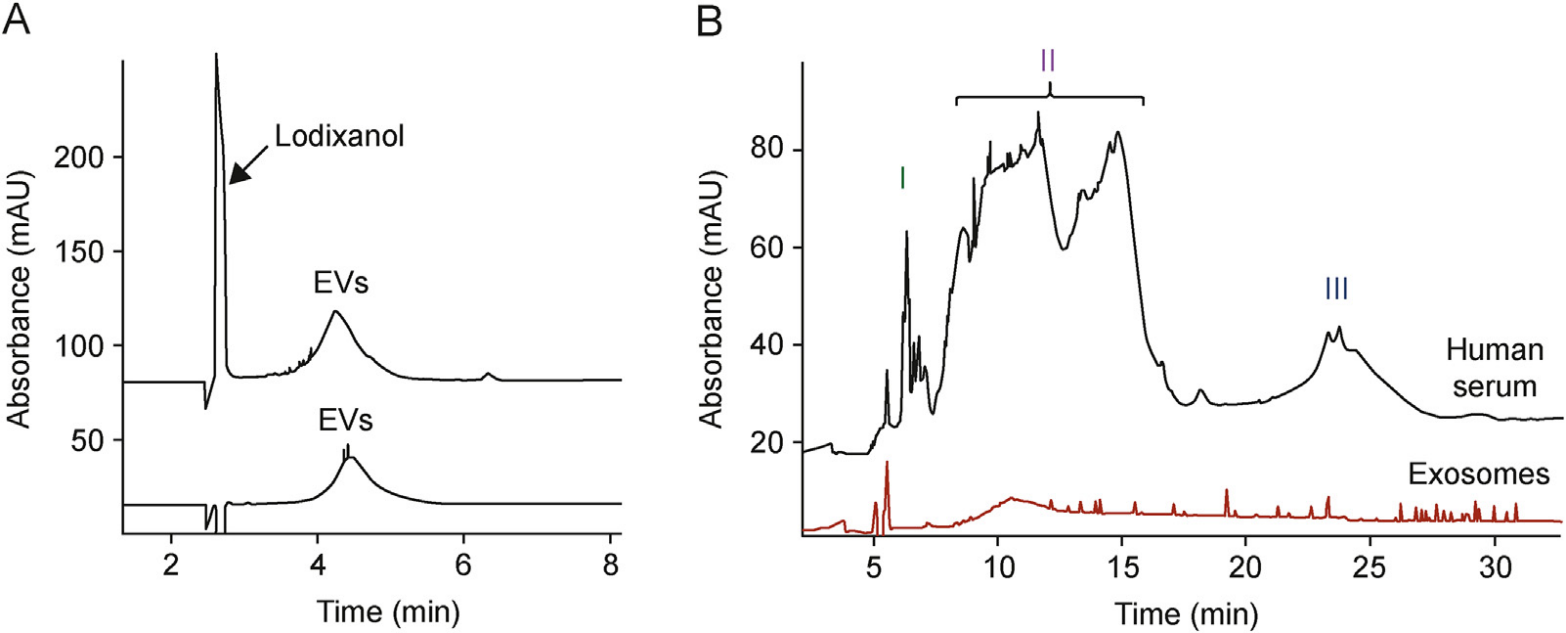
Analysis of extracellular vesicles (EVs) through capillary electrophoresis (CE). (A) CE analysis of the EVs isolated from the culture media of *Pectobacterium zantedeschia 9M*: (lower trace) wild-type and (upper trace) green fluorescent protein (GFP)-tagged strains [27]. (B) Results of the CE analyses of isolates obtained from human serum samples and standard exosomes [31]. I−III indicate groups of signals, featuring a specific ultraviolet (UV) spectrum, claimed to represent different types of EVs. Reprinted from Refs. [27,31] with permission.

In several studies, BGE was composed of 50-mM 1,3-bis(tris(hydroxymethyl)methylamino)propane (BTP) and 75-mM glycine (Gly) (pH 9.5) buffer [[25], [26], [27], [28], [29],^41^], as shown in Fig. 5A [27]. Large buffering counterions such as BTP have been proven to support the stability of nanoparticles during CE analyses owing to the steric effect [46,47]. Furthermore, the use of counter- and co-ions of similar pK_a_ values (BTP and Gly, respectively) increases the buffering capacity [26]. Morani et al. [48] used a similar concept. BGE consisting of Tris/*N*-cyclohexyl-2-aminoethanesulfonic acid (CHES) (pH 8.4; *I* = 90 mM) provided stable and symmetrical signals [30,42]. Morani et al. [30] have also reported that the increase in the *I* of a buffer from 50 to 150 mM enables the separation of residual dye from EVs without generating a high current (only 30 μA below 25 kV for *I* = 150 mM).

In another study, the application of borate and BTP/Gly buffers at three different *I* (8, 20, and 40 mM) for the analysis of bacterial EVs was explored. As the electric field increased, the *μ* of EVs decreased. In addition, the effect was substantially more pronounced when borate buffer was utilized. This could be attributed to the weaker screening of the surface charge of EVs by Na^+^ compared with BTP^+^ ions, resulting in greater *ζ*-potential of the vesicles and the occurrence of a relaxation effect. The decrease in *μ* with increasing electric field was smaller when higher *I* buffers were used, indicating the participation of EDL in the process (retardation effect) [41].

Similar observations were reported by Gao et al. [43]. The authors tested 10-mM phosphate buffer (pH 7.4) and various concentrations of phosphate-buffered saline (PBS; 0.1×, 0.5×, and 1.0×). When 10-mM phosphate buffer (pH 7.4) was used, a decrease in the *μ* of EVs was observed with a higher electric field strength. Paradoxically, the increase in PBS concentration (from 0.1× to 1×) resulted in a greater *μ* of the vesicles, which was accompanied by variations in the corrected peak areas. This, in turn, was caused by the instability of EVs under the investigated conditions due to the osmotic strength difference between the interior of the vesicles and BGE, as supported by TEM analysis. However, the authors reported that the EVs aggregate and that “potential vesicle debris resulting from EV deformation” may have been formed during sample preparation for microscopic analysis [43]. Thus, the explanation of the observed phenomenon needs further investigation.

Ouahabi et al. [31] used 0.1-M Tris and 0.25-M boric acid (pH 7.9) as BGE. According to the authors, the selected buffer prevented the aggregation of EVs and their adsorption on the inner wall of the uncoated capillary. Moreover, the authors proposed the addition of neutral linear polymer (hydroxypropyl cellulose (HPC)) and sodium dodecyl sulfate (SDS) to the BGE to reduce the adsorption of EVs to ionized silanol groups of the inner wall of the capillary. The addition of 0.1% (*m*/*V*) SDS to BGE was assumed to improve dispersion, prevent adsorption on the capillary walls, and enable the size-dependent separation of EVs through homogenization of the overall negative charge of the vesicle membranes [31]. The developed method was applied in the analyses of commercially available exosomes and isolates obtained from human serum samples (Fig. 5B) [31]. However, despite the good experimental conditions that the authors claimed, a low repeatability of the analyses was noted and no peak characteristic of EVs was observed.

### 2.4 Adsorption of EVs to the capillary wall

Adsorption of the analyte on the capillary wall in CE is usually observed as an effect of ion-ion interactions [49]. Because EVs are complex nanostructures containing proteins, lipids, nucleic acids, and small molecules, the deposition of these components on the inner wall of the capillary may lead to a loss of resolution, changes in the electroosmotic flow velocity, and repeatability issues [49].

Among the reviewed studies, only one discussed the adsorption of EVs to silica capillaries [31]. The problem was addressed by adding HPC and SDS as described in Section 2.3. In another interesting study, Morani et al. [30] compared the performance of bare fused silica and commercial, polyvinyl alcohol-coated capillaries during electrophoresis of bovine milk-derived EVs. The use of the modified capillary broadened the EV peak compared with the uncoated one. The same observations were made when poly(DMA-GMA-MAPS)-coated capillaries were used for the separation of OMVs from *Pectobacterium* sp. [41]. The signal broadening effect observed for the modified capillaries was proportional to the apparent migration velocity of vesicles, which is consistent with the theory of electrophoresis and excludes adsorption.

### 2.5 Detection

**Fig. 6:**
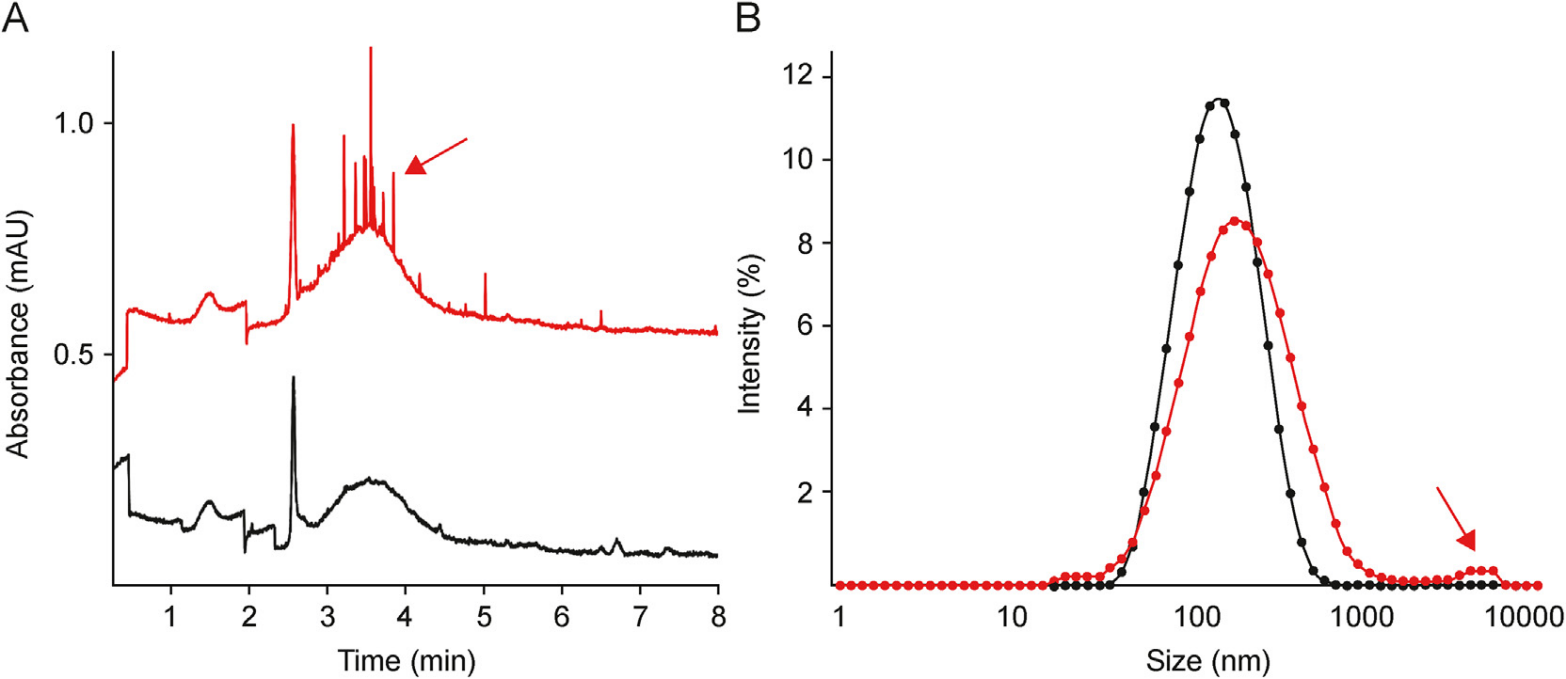
(A) Capillary electrophoresis (CE) and (B) dynamic light scattering (DLS) analyses of extracellular vesicles (EVs) isolated from *Citrus limon* using a combination of ultrafiltration (UF) and size-exclusion chromatography (SEC). Macromolecular aggregates are indicated by the red arrows. The black and red traces represent fractions 2 and 3 obtained through SEC during the isolation process [29]. Reprinted from Ref. [29] with permission.

In CZE, the signals generated by EVs are symmetrical with a relatively low separation efficiency (efficiency < 20,000 plates/m; Fig. 6 [29]). Various studies have proved that such signals are generated by dispersed nanoparticles of heterogeneous size as observed for inorganic particles, liposomes, and viruses [35,46,50]. These characteristic signals set EVs and other particles (e.g., lipoproteins or proteins) apart from low-molecular-weight compounds that typically migrate in narrow bands (efficiency > 100,000 plates/m).

#### 2.5.1 Ultraviolet (UV) detection

UV detection is the most universal type of detection and has been demonstrated in multiple studies to enable the detection of not only EVs but also various types of impurities [[25], [26], [27],^29^,^41^]. Piotrowska et al. [26] proposed the use of two wavelengths (200 and 230 nm) for EV detection. The authors used the 200-nm wavelength for vesicle quantification and the 230-nm wavelength (considered to be more selective) to confirm the identity of the peak [26]. Ouahabi et al. [31] utilized several wavelengths for the detection of EVs isolated from human blood serum samples. The authors were able to detect three groups of signals marked with I, II, and III in Fig. 5B [31], featuring a specific UV spectrum. Each of these groups represented different types of EVs. However, no additional analytical technique was employed for the characterization of the obtained isolates, which is why the detected signals remained unidentified. In addition, multiple signals in the electropherogram suggest the presence of a large number of impurities. Most of the marked signals do not have a characteristic EV shape, and the exosome standard is detected only in the area designated by the authors as II. Furthermore, no evidence of EV migration in regions I and III was provided.

Frequently, signals generated by EVs are accompanied by numerous peaks featuring extremely high separation efficiency (efficiency > 1,000,000 plates/m) and no specific *μ* [26,47]. This is because sequential analysis of the same sample results in the random detection of various numbers of such peaks. In the literature, these signals are referred to as “spikes”, and their appearance indicates the presence of aggregates [25,26,37]. Exemplary spikes are shown in Fig. 6 and are marked with a red arrow [29]. Similar spikes were observed in liposome formulations as an effect of their destabilization [51]. Aggregates of inorganic particles were also demonstrated to generate this type of peaks [52]. In such cases, the response of the UV detector is not a result of light absorption by solutes but is caused by light scattering by the detected objects [46,47]. Because the detection of spikes is random, it is impossible to quantify aggregates with CE. However, the increase in the injection length (injection volume) improves their detectability [41].

In two studies, UV detection was implemented for the simultaneous detection of impurities and EVs [25,27]. The EVs of bacterial origin from *Pectobacterium* were isolated using UC, UC with an iodixanol cushion, and iodixanol DGC. The use of iodixanol prevented EV damage during centrifugation and reduced the amount of observed macromolecular aggregates. Subsequently, CE enabled simultaneous monitoring of iodixanol residue and EV content in the isolates (Fig. 5A) [27].

#### 2.5.2 Laser-induced fluorescence (LIF) detection

EV staining is frequently performed in flow cytometry (FC) [53,54], NTA [55], Förster resonance energy transfer (FRET) [56], and microscopic analyses [57] and has also been recently implemented for EV detection by CE. The stainable structural components of EVs include lipids, nucleic acids, and proteins.

Morani et al. [30] used carboxyfluorescein diacetate succinimidyl ester (CFDA-SE) to stain EV proteins using the ability of this dye to permeate through cell membranes. After diffusing into the interior of the EVs, esterases cleaved CFDA-SE to form carboxyfluorescein succinimidyl ester (CFSE), which in turn interacted with amines, creating a highly fluorescent green dye. CFDA-SE does not cause any changes in the size or charge of EVs and does not generate fluorescent aggregates, a drawback that numerous lipophilic dyes have [53,58]. The detection limit obtained by the authors for EVs from whole bovine milk using the developed CE-LIF method was 8 × 10^9^ EVs/mL, and the calibration curve covered a range from 1.22 × 10^10^ to 1.20 × 10^11^ EVs/mL [30].

Tani and Kaneta [44] demonstrated the indirect detection and quantification of exosomes obtained from HeLa cell culture media using capillary electrophoretic immunoassay (CEIA). The authors measured the fluorescent anti-CD-63 antibody content before and after their offline incubation with EVs. Stoichiometric binding of EVs by antibodies was assumed, and a linear correlation between the amount of exosomes and the decrease in the signal area of the anti-CD-63 antibodies was demonstrated. However, although the application of antibodies for exosome quantification provided high selectivity to the method, it also limited its use to certain types of EVs because vesicles are known to differ in terms of the composition of their specific protein markers. For instance, EVs not involved in the endosomal pathway or directly formed and released from the plasma membrane may lack CD-63. Thus, the analysis of various types of EVs with CEIA requires the selection of proper antibodies [44].

**Fig. 7:**
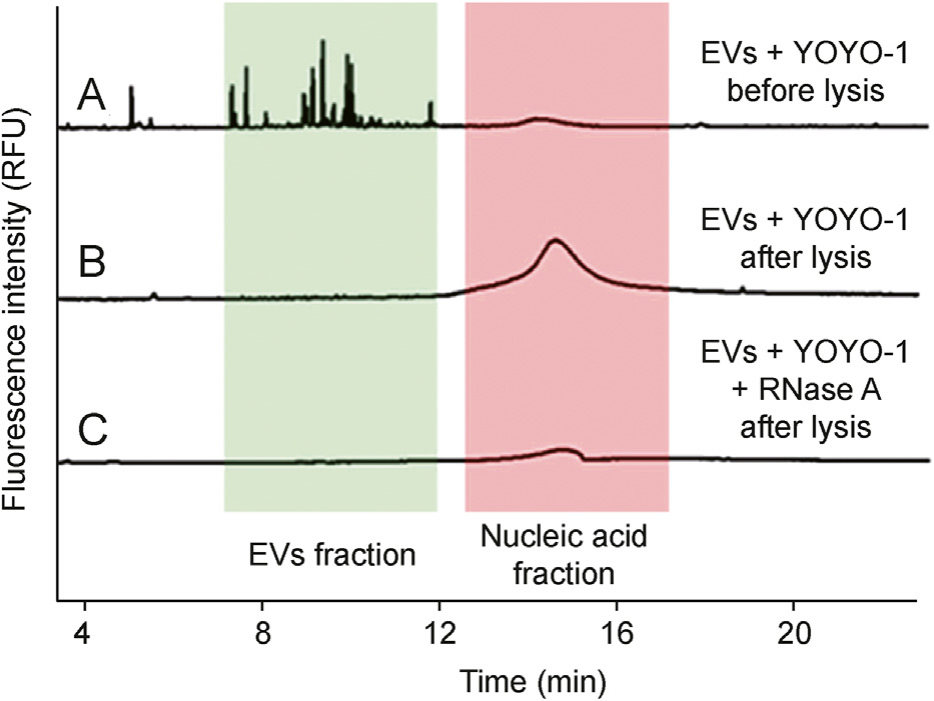
Capillary electrophoresis (CE) analysis of extracellular vesicles (EVs) isolated from MDA-MB-231 cell line culture medium [40]. (A) EV isolate directly stained with YOYO-1 dye. (B) EV isolate stained with YOYO-1 after lysis with 0.1% sodium dodecyl sulfate (SDS). The disappearance of EV-related signals and an increase in the nucleic acid peak are observed. (C) Sample B was treated with ribonucleases (RNase). The signal observed in the nucleic acid fraction represents the DNA content in the sample. RFU: relative fluorescence units. Reprinted from Ref. [40] with permission.

Dou et al. [40] used YOYO-1 to stain DNA and RNA carried by EVs. After staining, two zones were observed during the separation process: those characteristic of either free nucleic acids or EVs (Fig. 7) [40]. Free nucleic acid exhibited a broad peak in the electropherogram, whereas multiple narrow signals occurred in the region rich in EVs. As the selected dye did not permeate the EV membranes, SDS had to be used for lysis, resulting in the disappearance of signals in the EV region while the area of free nucleic acid peaks increased. The authors reported that the change in the peak area of the nucleic acids was proportional to the EV content in the analyzed sample. However, prior knowledge on nucleic acid content in a single vesicle was required for the quantification, limiting the applicability of the developed method to isolates devoid of various EV types.

LIF was used for the direct detection of bacterial EVs. Native *Pectobacterium* sp. strains were transformed with the plasmid encoding GFP which resulted in an increased secretion of EVs compared with the wild strain. Furthermore, CE-LIF analysis confirmed that GFP is incorporated into the secreted EVs [27].

Another work involves the use of CE for the analysis of EVs isolated from *Citrus limon* juice samples [29]. To confirm the identity of detected signals, the isolates were stained with SYBR Gold, a fluorescent dye selective toward nucleic acids [25]. The comparable separation efficiency, *μ*, and asymmetry of EV signals were demonstrated using the CE-UV and CE-LIF methods. Furthermore, no impact of SYBR Gold staining on the physicochemical properties of EVs was observed. The occurrence of peak asymmetry observed in some of analyzed samples was not related to the presence of impurities but was due to the heterogeneity of the EVs [29].

## 3 Comparison of CE with the recommended analytical techniques

Formulations containing EVs, dedicated for therapeutic use, are medicinal products [59]. To ensure the quality and safety of this novel class of biological drugs, manufacturers should establish standards for the quality control of formulations containing EVs. Comprehensive position statements and reports have recently addressed the issue of the manufacturing and characterization of vesicles and EV-related products [10,14,15,59]. Despite the general character of recommendations provided by the authors of these works, several important issues have been raised.

The preclinical phase of EV-based product development should include an exhaustive characterization of vesicles (used as active substances or drug carriers) and their formulations (finished product) [59]. The characterization of the product during the development phase should include physicochemical and immunochemical properties, purities, impurities, and biological activities [60]. At present, the characterization of EVs is performed according to the International Society for Extracellular Vesicles (ISEV) guidelines (published in 2023) and includes the measurement of the total protein, RNA, and lipid content in the samples (to estimate the concentration of vesicles), particle analysis (particle counting and analysis of their size distribution), electron microscopy (characterization of the EV morphology), and proteomic analysis (detection of EV proteomic markers and impurities) [15]. The data obtained during the development phase enables the selection of the most relevant parameters for product quality control [59]. It should be noted that multiparametric methods are favorable for more comprehensive screening of the isolates. For instance, NTA enables particle counting (quantitation of EVs) and simultaneously provides information on particle-size distribution (confirmation of the identity and assessment of purity). In the following section, the performance of CE is compared with ISEV-recommended techniques regarding the quality control of EV-based medicinal products.

### 3.1 Quantification

Total protein content assays (e.g., bicinchoninic acid assay (BCA)) and particle-counting techniques (e.g., NTA) are basic techniques used for EV quantification [12,14,61]. In addition to the numerous advantages of these commonly used techniques (e.g., relatively low cost per sample analysis and short measurement time), some serious drawbacks need to be considered. The commercial kits used for protein content measurements are not selective toward EVs, which may lead to an overestimation of vesicles because of the presence of proteomic impurities [62]. Furthermore, significant variability between different commercial tests has been reported [63]. The selectivity of typically used particle-counting techniques such as NTA or tunable resistive pulse sensing (TRPS) is also limited as these techniques are prone to picking up impurities, including protein aggregates and lipoproteins [8]. It has also been demonstrated that BCA and NTA are susceptible to the presence of polysaccharides used for culturing bacteria [25].

In general, FC does not show the problem related to low selectivity due to the application of immunofluorescent probes and well-established assay control [64]. It is currently becoming a gold standard in EV research owing to its high resolving capability, accuracy, and reproducibility [12,61,65]. As a single-particle detection technique, FC provides straightforward information on the number of vesicles in the analyzed sample, which outperforms techniques dependent on bulk detection such as CE. However, separation and detection upon CE analysis are not limited by the size of the solute, whereas the detection of nonfluorescent particles < 100 nm by FC is technically limited [64]. Although no study comparing CE and FC has been conducted, it might be assumed that combining these two techniques may provide more comprehensive quantitative and qualitative information.

Nevertheless, a correlation between protein content (as determined through BCA), particle concentration, and peak area obtained during CE analysis has been demonstrated in several studies [25,26,29,30]. This includes isolates obtained from bacterial and eukaryotic cell culture media, plant materials, and bovine milk samples. Both UV and LIF detections were found to be suitable in this context, and the studies highlighted the importance of a good quality of isolates when a less selective UV detector is used for analysis. Optimization of the methodology used for the isolation of bacterial EVs by implementing the use of iodixanol cushioning and DGC instead of direct UC proved to be advantageous in terms of not only the yield but also of improving the correlation between the results of the CE analysis and protein content assay [25]. The influence of the quality of the isolate on the correlation between the methods used for the quantification of vesicles has also been observed for plant-derived EVs [25,29].

### 3.2 Purity

The presence of impurities is typically assessed based on the ratio of particle number to total protein content (particles to protein ratio (PtP)), a concept originally proposed in 2013 by Webber and Clayton [66]. Despite the fact that this parameter can indicate the presence of only a certain type of impurities, the PtP is frequently used in the literature and recommended in position statements [14,59]. However, PtP has been shown to have poor correlation with the high-resolution analysis of EV surface markers [67]. The ISEV guidelines suggest that lipid/particle or lipid/protein ratios should be reported for a more reliable evaluation of the EV quality [14]. The recommended assays are easily applicable and provide information on the general quality of isolates; however, the identity of impurities remains unknown unless an additional analytical technique is employed. Although proteomic contaminants (so-called negative markers) can be detected through Western blot (WB) or enzyme-linked immunosorbent assay (ELISA), it needs to be emphasized that the overall usability of targeted analysis for profiling impurities is strongly limited [12]. While the implementation of mass spectrometry (MS) may be useful, its applicability in routine analysis remains limited due to the high costs [8,14,61].

CE enables the simultaneous separation of small molecules, proteins, and micro-/nano-particles, which allows for the screening of various classes of impurities, including process-related impurities. The detection of macromolecular aggregates and determination of iodixanol (small molecule) together with EVs [26,27,30] has been discussed in Section 2.5. In another study, Morani et al. [42] were able to detect the bleeding of commercially available poly-l-lysine-coated beads (ExoCAS-2) used for EV isolation.

**Fig. 8:**
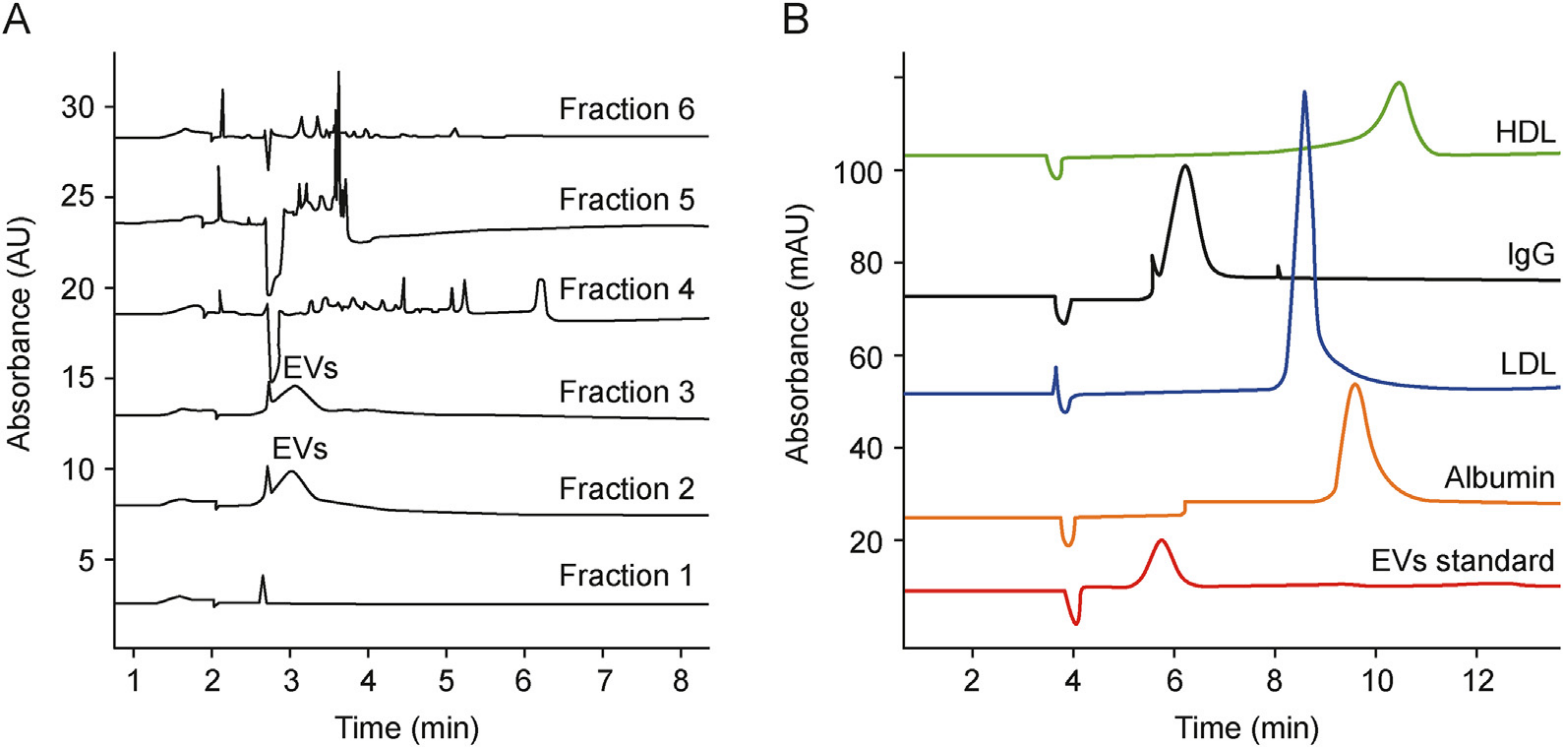
Capillary electrophoresis (CE) separation of extracellular vesicles (EVs) and sample matrix components. (A) CE analysis of seven fractions obtained through size-exclusion chromatography (SEC) separation of *Citrus limon* juice samples [29]. (B) Comparison of high-density lipoproteins (HDL), immunoglobulin G (IgG), low-density lipoproteins (LDL), albumin, and exosome (EV standard) separation [43]. Reprinted from Refs. [29,43] with permission.

Host cell proteins and DNA are important classes of process-related impurities [60]. Recently, the use of CE has enabled monitoring of the isolation of EVs from Chinese hamster ovary (CHO) cell culture media and *Citrus limon* juice samples [28,29]. The technique not only distinguished between SEC fractions containing vesicles and those free of vesicles but also clearly demonstrated that none of the impurities observed in the latter were present in the EV-rich fractions (Fig. 8A) [29]. Gao et al. [43] applied CE as a two-dimensional (2D) technique for the separation of human serum sample components. CE was proved to be capable of separating EVs from IgG, albumin, and lipoproteins (Fig. 8B) [43]. In Section 2.5.2., the detection of extravesicular and EV-carried nucleic acids has been discussed [29,40]. Thus, the ability of CE to detect host cell proteins and free DNA meets the requirements of the European Medicines Agency [60].

Notably, complete removal of process-related impurities from EV-based medicinal formulations is not recommended at all cost [59]. It has been shown that a protein corona around the EV resulting from the presence of proteins in the sample may positively affect the stability and potency of EVs [68,69]. Thus, instead of excessive purification of the EVs to remove proteins, characterization of these impurities, evaluation of batch-to-batch consistency, and determination of acceptable upper limits are recommended [10,59]. Because CE is routinely used in impurity profiling of small molecular and biological drugs, its application for the purity analysis of EV-based pharmaceuticals in this context should be considered at the product development stage [70].

### 3.3 Identification

Identity confirmation of EVs is achieved using a combination of TEM, proteomic, and particle-size distribution analysis [14,15,61]. This approach enables the visualization of isolated structures and confirmation of the presence of selected markers. Moreover, the implementation of immunogold electron microscopy undeniably confirms the presence of certain markers on the surface of obtained EVs. Although this confidence level is not achievable with any other technique, the application of CE offers some unconventional possibilities in this context.

**Fig. 9:**
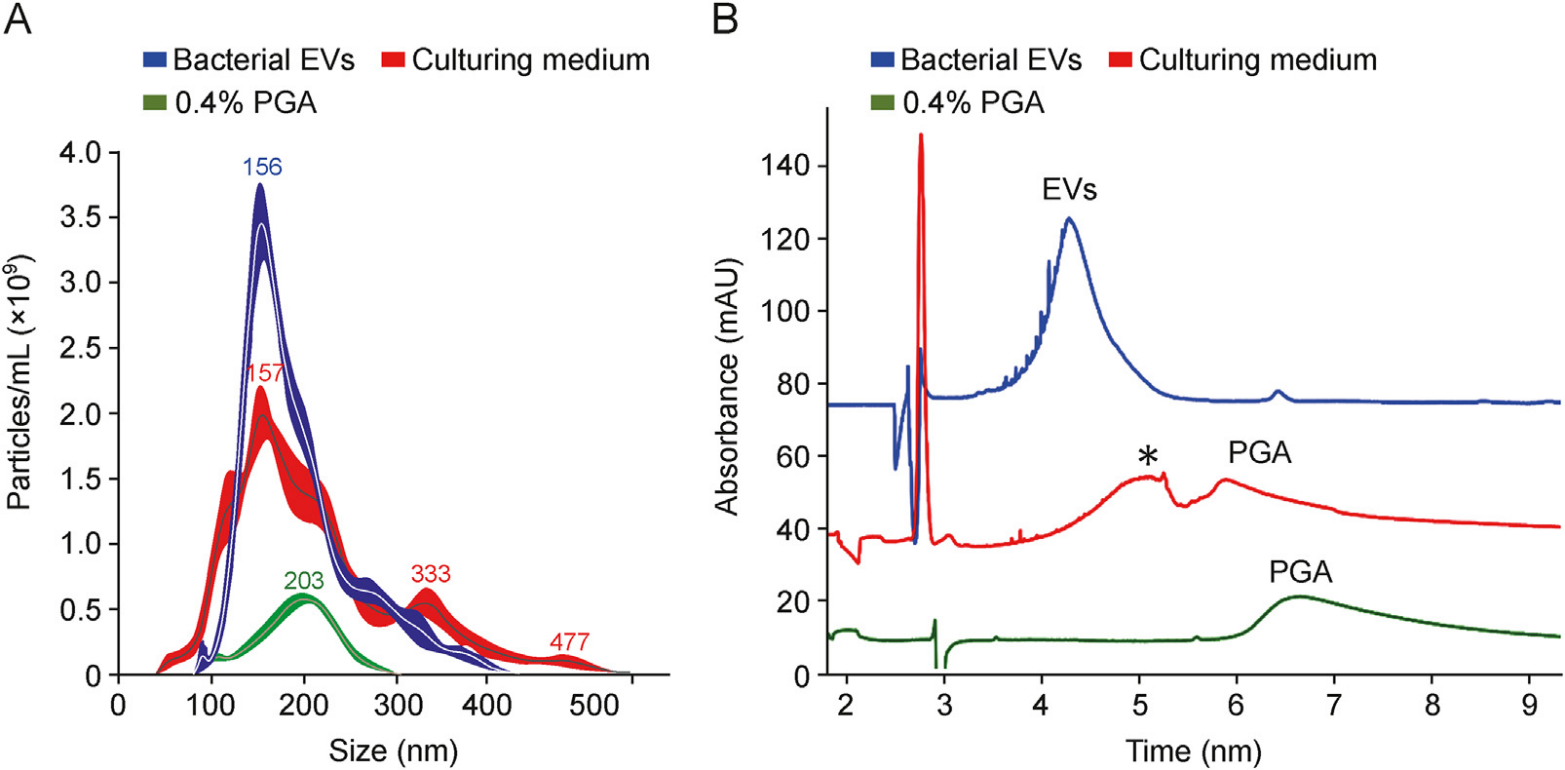
Comparison of (A) nanoparticle tracking analysis (NTA) and (B) capillary electrophoresis (CE) analysis results of isolates obtained from *Pectobacterium zantedeschia* culture (blue traces), culture medium composed of M63 medium, 0.2% glycerol and 0.4% poly(galacturonic acid) (PGA) (red traces), and 0.4% PGA solution (green traces) [25]. ∗ Indicates an unidentified component of the culture medium isolate. Reprinted from Ref. [25] with permission.

The separation of compounds by CZE is dependent on the charge-to-size ratio. Certain vesicles are expected to exhibit characteristic *μ* values, differentiating them from other components of the analyzed sample. This property can be used for purity assessment and preliminary confirmation of compound identity. Gao et al. [43] reported that CE is able to separate exosomes from common impurities co-isolated from human blood (Fig. 8B [43]). In another work, the differences in the *μ* values of the detected compounds distinguished EVs from poly(galacturonic acid) (PGA), which was used as a bacteria culture medium component (Fig. 9) [25]. Furthermore, *μ* measurements may be used to assess EV quality because the physicochemical properties of EVs were reported to vary as a result of vesicle breakdown over time [71]. Nevertheless, measurement of the *μ* of EVs alone is insufficient for clear confirmation of the identity of the separated compound.

Chemically, vesicles are a mixture of proteins, lipids, and numerous other organic and inorganic molecules, such as nucleic acids [72]. Although the exact composition of particular EV types remains a subject of research, selective staining of certain components of vesicles is commonly employed in FC and has already been demonstrated in CE, as discussed in Section 2.5.2. In this context, the application of selective dyes and molecular probes facilitates the screening of vesicle cargo, which, together with the high selectivity of CE, is advantageous in studies on EV subpopulations.

## 4 Future perspectives

**Fig. 10:**
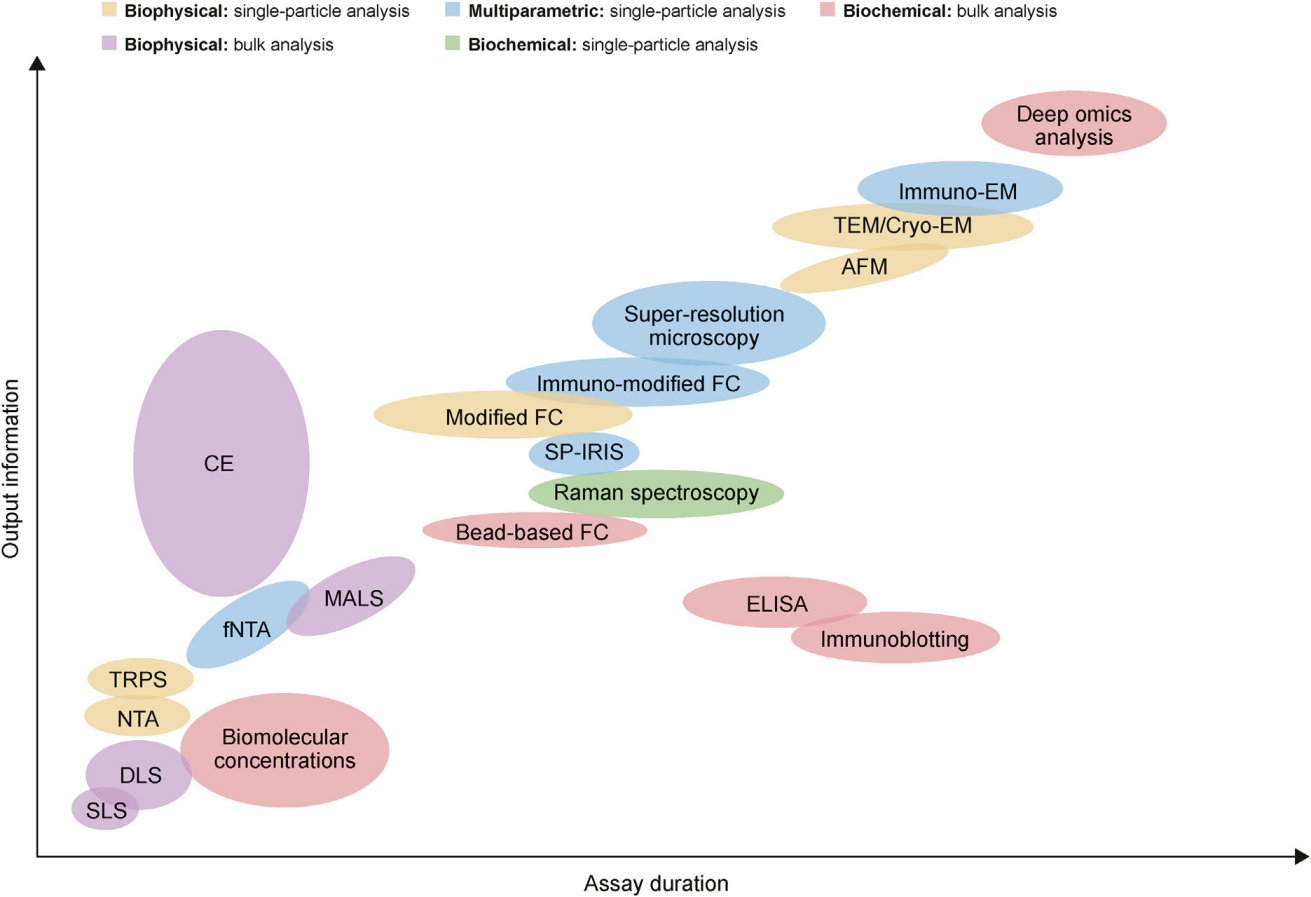
Comparison of different analytical techniques used for extracellular vesicle (EV) characterization with capillary electrophoresis (CE) in relation to the time and output information obtained using specific assays [61]. CE analysis usually takes about 20–30 min (including capillary conditioning) and provides various information on vesicle quantity, purity, heterogeneity, and identity. SLS: static light scattering; DLS: dynamic light scattering; NTA: nanoparticles tracking analysis; TRPS: tunable resistive pulse sensing; fNTA: fluorescent nanoparticles tracking analysis; MALS: multiangle light scattering; FC: flow cytometry; ELISA: enzyme-linked immunosorbent assay; SP-IRIS: single-particle interferometric reflectance imaging sensors; AFM: atomic force microscopy; TEM/Cryo-EM: transmission/cryo-electron microscopy (EM). Reprinted from Ref. [61] with permission.

CE enables the quantification of EVs, provides qualitative information, and offers high-performance separation of a great variety of analytes, making it a perfect tool for the screening of impurities. The utility of CE in EV heterogeneity assessment has already been demonstrated [25,29]. These advantages make CE suitable for the detection of both product- and process-related impurities in EV manufacturing, regardless of the production scale. Notably, CE analyses consume a relatively small amount of time and can be performed with no or minimal sample preparation. In total, CE offers an attractive ratio between the amount of delivered information and the analysis time (Fig. 10) [61]. Currently, the concept of employing CE for EV characterization is still at an early stage. The use of CE is currently marginal when compared with the total number of articles published each year on this topic (Fig. 1 and Table 2). However, the importance of CE is expected to increase in the coming years mainly due to the demand of the industry for reliable tools for quality control of EV-based therapeutic products. Before this happens, however, the concept of using CE for EV characterization needs to undergo further testing.

EVs of various origins are within the scope of interest of both pharmaceutical and medicinal research [10]. Among all vesicle sources investigated to date, EVs derived from mesenchymal stem cells have gained the main attention owing to their regenerative properties [4]. However, to date, no study on the characterization of EVs isolated from mesenchymal stem cell cultures with CE has been published. Thus, assessment of the usability of CE for the characterization of this type of EVs is considered to be desirable. Furthermore, the development of standardized protocols for the characterization of EVs isolated from clinically relevant sources (e.g., human plasma, serum, or urine) is valuable.

It is estimated that the largest global EV market share (about 45%) is held by kits and reagents used for EV characterization and isolation [73]. It is predicted that such a high share will be sustained in the next few years, which shows the high demand for such products. Thus, the development of commercial kits dedicated to specific applications may be groundbreaking for the routine use of CE in EV research.

However, the developed kits and protocols will need to undergo a validation process. To date, the validation of methods based on different techniques used for EV characterization, including CE, has been strongly limited by the poor availability of vesicle standards. Consequently, published CE validation studies are scarce and often limited to linearity and precision assessment [25,26,29,30,43,44]. The mentioned articles address this issue by comparing the results obtained using CE and other techniques, such as NTA or BCA. However, correlations between the use of CE and other techniques have been shown to be dependent on the material used for EV isolation and the purity of the analyzed samples [25,29]. Nevertheless, purified vesicles of different origins or even reference standards are currently being offered more often by commercial vendors.

Analysis of complex structures such as EVs poses difficulties but also creates possibilities. For instance, immunolabeling or selective staining of certain vesicle components is common practice in FC analysis, which not only enables quantification of EVs but also provides qualitative information. Furthermore, initial reports on CE analysis of stained EVs have already been published [29,30], but more advanced staining strategies in CE have not yet been implemented. In particular, the application of smart and specific nanoprobes may facilitate the detection of certain types or subpopulations of EVs [[74], [75], [76]]. The application of more specific probes may also create real alternatives to WB analysis and substantially contribute to the dissemination of CE in EV research. Furthermore, CE exhibits great potential for use in the identity confirmation of EVs in validated EV isolation processes. Such an identification strategy may be based on multiple criteria, including the measurement of μ and the analysis of samples stained with one or several dyes/probes.

Although it has not yet been investigated, analyzing the impact of the EV corona on μ may be relevant (Section 2.2.2), as CE has proved to be capable of monitoring the interplay between proteins and synthetic nanoparticles *in vitro* [77]. Considering the importance of corona components for EV biological activity or stability, CE analysis would be useful in studying these interactions.

## 5 Conclusion

The rapid development of EV-derived medicinal products is expected to revolutionize drug delivery and regenerative medicine. Assurance of the safety of this novel class of biological drugs requires the development of efficient analytical strategies for product quality control. Because of the complexity and heterogeneity of EVs, the application of currently recommended techniques may not be sufficient to meet the stringent criteria of the regulator for the purity assessment. Currently available literature shows that CE can overcome these problems by providing lacking information, complementary to those obtained with FC. Hence, the use of CE should be considered during the preclinical phase of product development. However, it needs to be emphasized that the concept of using CE for the characterization of EVs is new, and substantial developments in this field are expected in the coming years.

## CRediT authorship contribution statement

**Aleksandra Steć:** Writing – original draft, Visualization, Investigation, Funding acquisition, Conceptualization. **Andrea Heinz:** Writing – review & editing, Validation, Supervision, Funding acquisition, Conceptualization. **Szymon Dziomba:** Writing – original draft, Visualization, Supervision, Investigation, Funding acquisition, Conceptualization.

## Declaration of competing interest

The authors declare that there are no conflicts of interest.
