# Supplementary material for: Characterization of extracellular vesicles by capillary zone electrophoresis: A novel concept for characterization of a next-generation drug delivery platform
Source: J Pharm Anal. 2024 May 23;14(12):101004. doi: 10.1016/j.jpha.2024.101004 (PMC11721263; doi:10.1016/j.jpha.2024.101004)
Supplement: Multimedia component 5 [file mmc5.pdf]

CCC RightsLink

SD

ACS Publications
Most Trusted. Most Cited. Most Read.

Offline Coupling of Asymmetrical Flow Field-Flow Fractionation and Capillary Electrophoresis for Separation of Extracellular Vesicles

Author: Ziting Gao, Zachary Hutchins, Zongbo Li, et al

Publication: Analytical Chemistry

Publisher: American Chemical Society

Date: Oct 1, 2022

Copyright © 2022, American Chemical Society

PERMISSION/LICENSE IS GRANTED FOR YOUR ORDER AT NO CHARGE

This type of permission/license, instead of the standard Terms and Conditions, is sent to you because no fee is being charged for your order. Please note the following:

- Permission is granted for your request in both print and electronic formats, and translations.
- If figures and/or tables were requested, they may be adapted or used in part.
- Please print this page for your records and send a copy of it to your publisher/graduate school.
- Appropriate credit for the requested material should be given as follows: "Reprinted (adapted) with permission from {COMPLETE REFERENCE CITATION}. Copyright {YEAR} American Chemical Society." Insert appropriate information in place of the capitalized words.
- One-time permission is granted only for the use specified in your RightsLink request. No additional uses are granted (such as derivative works or other editions). For any uses, please submit a new request.

If credit is given to another source for the material you requested from RightsLink, permission must be obtained from that source.

BACK CLOSE WINDOW

© 2024 Copyright - All Rights Reserved | Copyright Clearance Center, Inc. | Privacy statement | Data Security and Privacy | For California Residents | Terms and ConditionsComments? We would like to hear from you. E-mail us at customercare@copyright.com

Prywatność - Warunki
